# Effect of D-cysteine on dual-species biofilms of *Streptococcus mutans* and *Streptococcus sanguinis*

**DOI:** 10.1038/s41598-019-43081-1

**Published:** 2019-04-30

**Authors:** Xiao Guo, Shiyu Liu, Xuedong Zhou, Hongying Hu, Keke Zhang, Xinmei Du, Xian Peng, Biao Ren, Lei Cheng, Mingyun Li

**Affiliations:** 1 State Key Laboratory of Oral Diseases & National Clinical Research Center for Oral Diseases, West China Hospital of Stomatology, Sichuan University, Chengdu, 610041 China; 2 Department of Cariology and Endodontics, West China Hospital of Stomatology, Sichuan University, Chengdu, 610041 China; 3 Institute of Stem Cell and Tissue Engineering, School and Hospital of Stomatology, Wenzhou Medical University, Wenzhou, 325035 China

**Keywords:** Bacterial development, Biofilms

## Abstract

Dental caries is a highly prevalent disease worldwide. It is caused by the cariogenic biofilms composed of multiple dynamic bacteria on dental surface. *Streptococcus mutans* and *Streptococcus sanguinis* are resident members within the biofilms and an antagonistic relationship has been shown between these two species. *S. mutans*, as the major causative microorganism of dental caries, has been reported to be inhibited by free D-cysteine (D-Cys). However, whether D-Cys could affect *S. sanguinis* and the interspecies relationship between *S. mutans* and *S. sanguinis* remains unknown. The aim of the current study was to investigate the effect of D-Cys on the growth and cariogenicity of dual-species biofilms formed by *S. mutans* and *S. sanguinis*. We measured dual-species biofilms biomass, metabolic activity, lactate production. We also detected the biofilms structure, the ratio of live/dead bacteria, extracellular polysaccharide (EPS) synthesis and bacterial composition in the dual-species biofilms. We found that D-Cys could reduce the metabolic activity and lactic acid production of dual-species biofilms (*p < *0.05). In addition, biofilms formation, the proportion of *S. mutans* in dual-species biofilms, and EPS synthesis were decreased with D-Cys treatment. The results suggested that D-Cys could inhibit the growth and cariogenic virulence of dual-species biofilms formed by *S. mutans* and *S. sanguinis*, indicating the potential of D-Cys in clinical application for caries prevention and treatment.

## Introduction

Dental caries is one of the most major and prevalent diseases worldwide^1^. According to the statistics of the United States from 2011 to 2012, approximately 90% of U.S. adults aged 20–64 had dental caries in permanent teeth^2^. Dental plaque composed of complex multiple bacteria is the major cause of dental caries^3^. *Streptococcus mutans* is an important member in the dental plaque and the key contributor to tooth demineralization owning its ability of adhesion to dental surface, acid production, acid tolerance and exopolysaccharides (EPS) synthesis^4–6^. While *Streptococcus sanguini*s is another indigenous species in the dental plaque. An antagonistic relationship has been shown between *S. mutans* and *S. sanguinis*. The mutacin produced by *S. mutans* could inhibit *S. sanguinis* growth^7^, while *S. sanguinis* could produce hydrogen peroxide (H_2_O_2_) to inhibit the initial biofilm formation of *S. mutans*^8^. Moreover, early colonization of *S. sanguinis* to dental surface delayed the colonization of *S. mutans* to tooth^9^.

D-amino acids (D-AAs), as the component of cell wall peptidoglycan^10,11^, were demonstrated to participate in regulating and disassembling bacterial biofilms. For this reason, D-AAs has been assumed to provide a new strategy for the prevention of biofilm-related diseases^12,13^. Tong Z. *et al*.^14^ indicated that free D-cysteine (D-Cys) could restrain the biofilms formation of *S. mutans*. Since *S. mutans* is antagonistic to *S. sanguinis*, whether D-Cys could affect *S. sanguinis* and the interspecies relationship between *S. mutans* and *S. sanguinis* remains unknown. Therefore, the present study was aimed to investigate the effect of D-Cys on dual-species biofilms formed by *S. mutans* and *S. sanguinis*. We hypothesized that: (1) D-Cys was able to inhibit the growth and metabiotic activity of the dual-species biofilms; (2) D-Cys could regulate the proportion of *S. mutans* and *S. sanguinis* and convert the biofilm to a healthier condition.

## Results

### Growth of the *S. mutans* and *S. sanguinis* affected by D-cysteine

**Figure 1:**
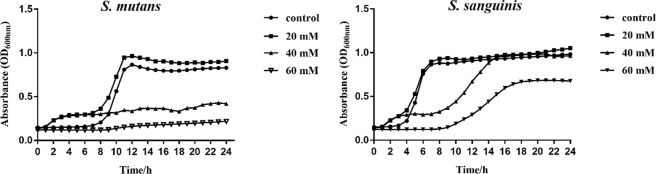
Growth curve of *S. mutans* and *S. sanguinis* affected by different concentrations of D-Cys for 24 h.

As shown in Fig. 1, D-Cys inhibited the growth of *S. mutans* at concentrations of 40 mM and 60 mM. The D-Cys hardly affected the growth of *S. sanguinis* at 20 mM. At the concentration of 40 mM, the D-Cys inhibited the growth of *S. sanguinis* at first, but it gradually recovered normal growth after 12 h. At the concentration of 60 mM, the D-Cys slowed down the growth of *S. sanguinis* in some extent.

### D-Cys inhibited Biofilms formation and metabolic activity

**Figure 2:**
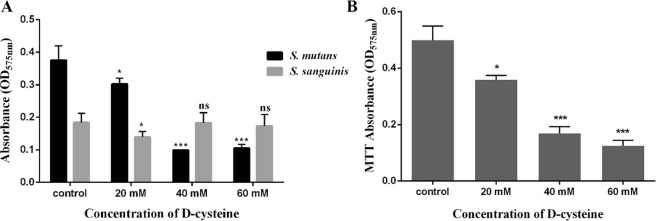
Biofilm biomass of single-biofilms (**A**) and metabolic activity of dual-species biofilms (**B**) affected by different concentrations of D-Cys. The data are presented as the means + standard deviation from three independent experiments, and the asterisks represent significant differences compared with the non-treated control group (*P < 0.05, ***P < 0.001, ns: no significance).

Biofilm biomass of the single species was measured. As the data shown in Fig. 2A, D-Cys significantly inhibited biofilms formation of *S. mutans*. Biofilm biomass decreased in all D-Cys treatment groups. However, D-Cys slightly influenced biofilms formation of *S. sanguinis*. Biofilm biomass decreased a bit in 20 mM group, and no significant difference was found in 40 mM and 60 mM groups compared to the non-treated control group. The result of metabolic activity of dual-species biofilms affected by D-Cys was shown in Fig. 2B. Biofilms in control group displayed a relatively high metabolic activity. Biofilms metabolic activity were significantly decreased with D-Cys treatment, especially in 40 mM and 60 mM groups.

### D-Cys inhibited lactic acid production in dual-species biofilms

**Figure 3:**
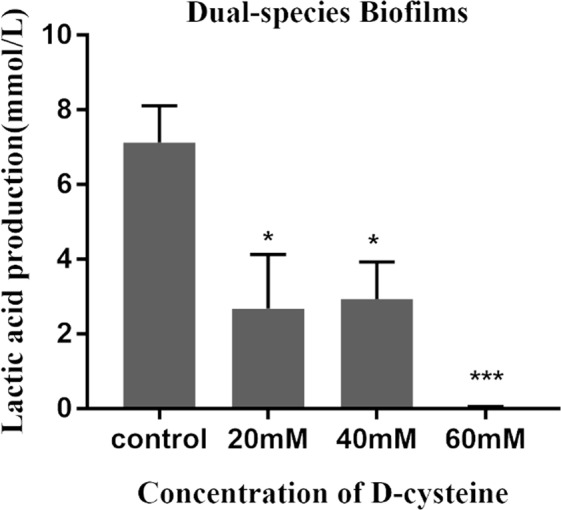
Production of lactic acid in dual-species biofilms affected by D-Cys. The data are presented as the means + standard deviation from three independent experiments, and the asterisks represent significant differences compared with the non-treated control group (^*^P < 0.05, ^***^P < 0.001).

Acid excreted by cariogenic bacteria is the direct cause of tooth demineralization. We examined lactic acid production in dual-species biofilms (Fig. 3). 20 mM and 40 mM of D-Cys markedly inhibited acid production. Lactic acid production in these two groups was approximately 1/3 of that in the control group. Besides, lactic acid was hardly to be detected in 60 mM of D-Cys group.

### D-Cys decreased the ratio of live/dead cells in dual-species biofilms

**Figure 4:**
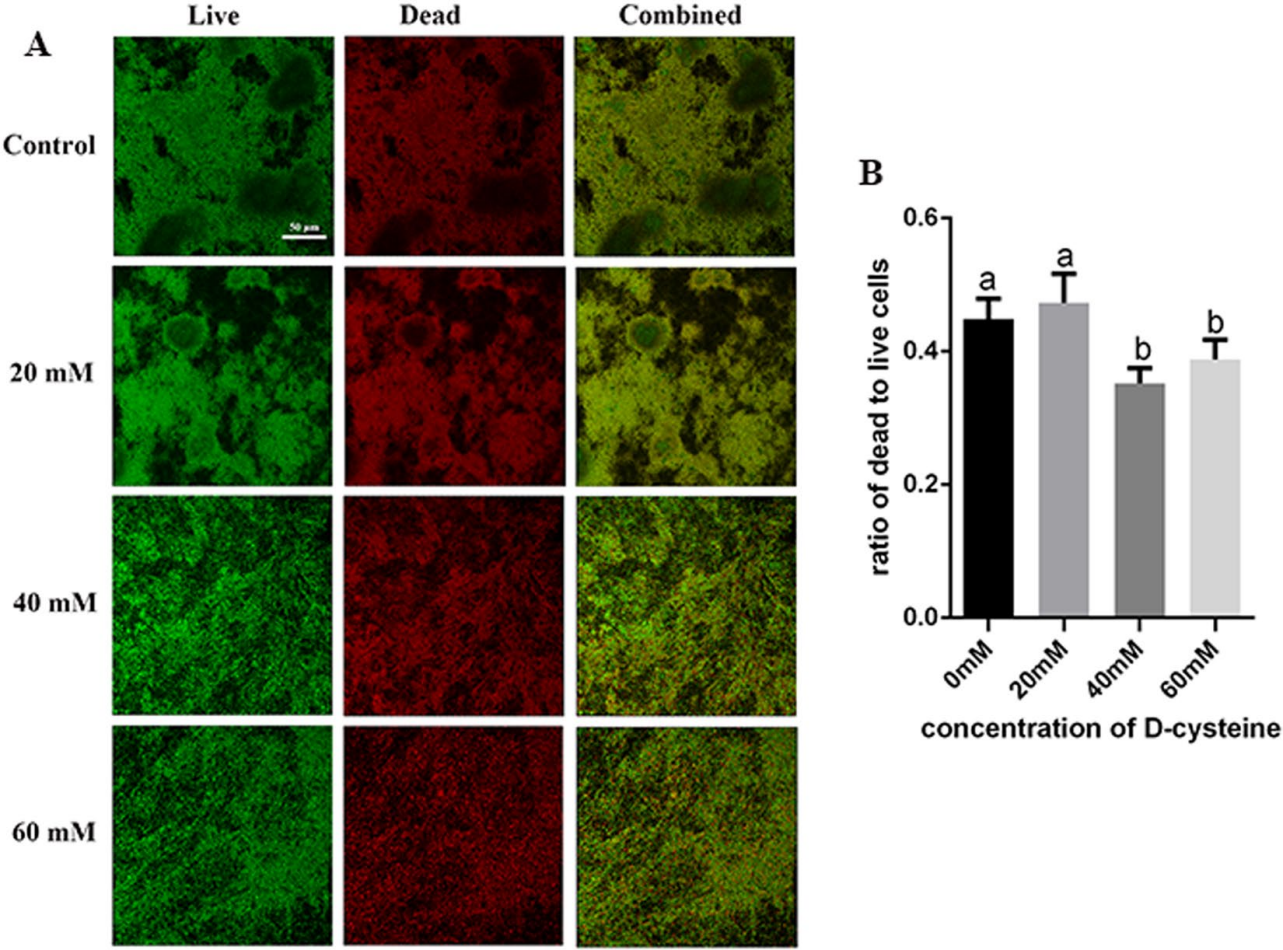
Live/Dead bacteria staining assay of dual-species biofilms. (**A**) Live bacteria were stained green and dead bacteria were stained red. (**B**) The ratio of live/dead bacteria computed in line with 3 random sights of dual-species biofilms. The data are presented as the means + standard deviation. In each plot, different letters mean significance between the two groups (^a&a^no significance, ^a&b^*P* < 0.05).

Live bacteria were stained green and dead bacteria were stained red. The ratio of live/dead cells was decreased in 40 mM and 60 mM groups (Fig. 4), indicating that the proportion of live cells was up-regulated by D-Cys. However, the bacterial composition of the live cells was unclear.

### D-Cys inhibited the EPS synthesis in dual-species biofilms

**Figure 5:**
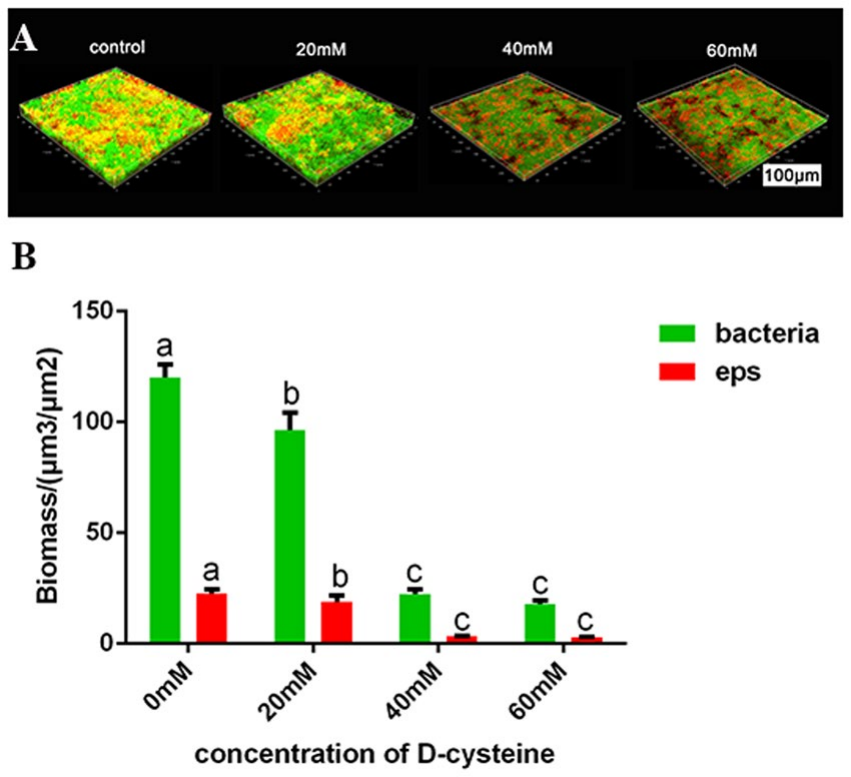
Extracellular polysaccharides (EPS) of dual-species biofilms. (**A**) The 3-dimensional reconstruction of dual-species biofilms (bacteria, stained green; EPS, stained red). (**B**) The volume of EPS and bacteria, calculated according to 3 random sights of biofilms (mean + sd). In each plot, different letters mean significance between the two groups (^a&b^P < 0.05, ^a&c^P < 0.001).

It was reported that the EPS are the key cariogenic virulence of *S. mutans*^15^. We measured EPS synthesis by confocal laser scanning microscopy (CLSM). Both bacterial cell (green) numbers and EPS (red) synthesis were decreased by D-Cys (Fig. 5B). Biofilms were looser and less EPS were around the bacteria in three D-Cys-treated groups (Fig. 5A).

### D-Cys disassembled bacterial biofilms

**Figure 6:**
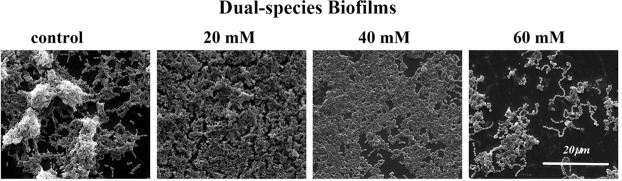
Scanning electron microscopy (SEM) micrographs of dual-species biofilms structure.

The structure of biofilms was imaged by a scanning electron microscopy (SEM). D-Cys disassembled bacterial biofilms (Fig. 6). Biofilms structures were more and more loose and less bacterial clusters were observed along with the rise of D-Cys concentration. The complete structure of biofilms disappeared and there were many aperture gaps in 60 mM group.

### D-Cys up-regulated the proportion of *S. sanguinis* cells in dual-species biofilms

**Figure 7:**
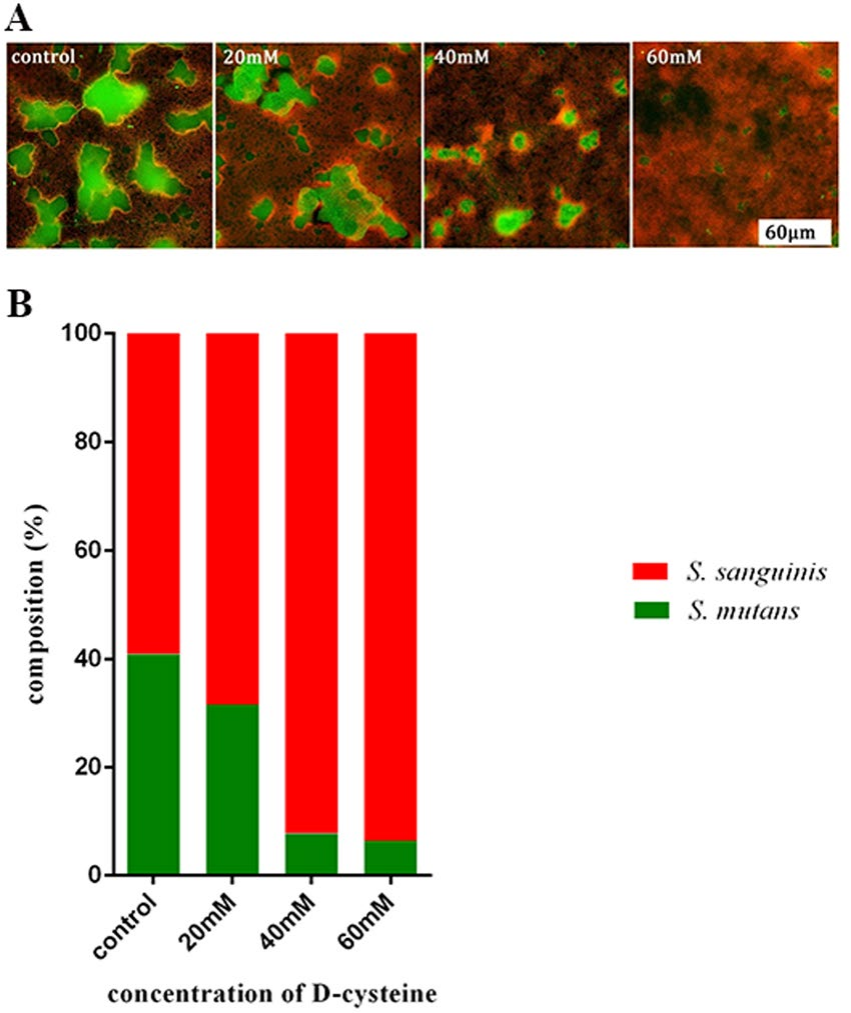
(**A**) Fluorescent *in situ* hybridization images of dual-species biofilms (*S. mutans*, stained green; *S. sanguinis*, stained red). (**B**) Bacterial composition in dual-species biofilms.

The species-specific fluorescent *in situ* hybridization (FISH) labeled biofilms were imaged and quantitative real-time polymerase chain reaction (q-PCR) was performed to analyze the bacterial proportion in dual-species biofilms. *S. mutans* cells were labeled green and *S. sanguinis* cells were labeled red. As shown in Fig. 7A, the integrated green fluorescence intensity was much weaker and integrated red fluorescence intensity was much stronger in D-Cys-treated groups, indicating a down-regulation of *S. mutans* and an up-regulation of *S. sanguinis* in dual-species biofilms. According to the results of q-PCR, the ratio of *S. mutans* to *S. sanguinis* was appreciably decreased by D-Cys (Fig. 7B). D-Cys altered the bacterial composition in dual-species biofilms, making the proportion of *S. sanguinis* higher.

## Discussion

Recent studies have revealed that D-Cys could inhibit the biofilm formation of *S. mutans*. However, dental caries is a disease caused by complex microflora composed of multiple bacteria. *S. mutans* and *S. sanguinis* are predominant members in the dental plaque. In the present study, we investigated the effect of D-Cys on the growth and cariogenicity of dual-species biofilms formed by *S. mutans* and *S. sanguinis*. The results indicated that D-Cys significantly inhibited biofilms formation, biofilms activity, lactic acid production, and EPS synthesis. Meanwhile, D-Cys altered the proportion of *S. mutans* and *S. sanguinis* in dual-species biofilms.

Biofilms provided important living space for bacteria^16–18^, and could help bacteria escape from the host defenses and resist the harmful factors^19–21^. Considering the importance of biofilms for bacteria, we detected whether D-Cys could affect dual-species biofilms formation. By the SEM observation, we found D-Cys appreciably disassembled bacterial biofilms, biofilm structure was incomplete in 60 mM of D-Cys group. We interestingly found the D-Cys at concentration of 20 mM might slightly promote the growth of planktic bacteria (Fig. 1), but it inhibited the single-species biofilm formation. We guessed the lower concentration might promote the growth of *S. mutans* and *S. sanguinis*. When we tested the biofilm formation by crystal violet staining assay, we need to wash the loose bacteria and it led the planktonic bacteria washed away. Although the growth curve of 20 mM seemed higher than the control group, there was no statistical difference.

The bacteria in the dental plaque could metabolize carbohydrates (primarily sucrose) to acids, causing the demineralization of tooth surface^22,23^. Biofilm activity and lactic acid production are associated with the cariogenic capacity. In the present study, we found that D-Cys significantly inhibited biofilms activity and decreased lactate production, indicating a down-regulation of biofilms cariogenicity by D-Cys. The decrease of lactic acid production might result from the inhibition of D-Cys to the dual-species biofilms formation. EPS, the major component in the biofilms, were also the important virulence of the biofilms^24^. EPS can help bacteria adhere to tooth surfaces, provide protection from the outside pressure and provide nutrition, which supply a suitable environment for microbes^25^. The result of confocal laser scanning microscopy assay showed that D-Cys significantly reduced the total synthesis of EPS.

Clinic research has showed that dental caries status in children, the interaction of *S. mutans* with *S. sanguinis*  was an important factor^26^. Therefore, proportion of *S. mutans* and *S. sanguinis* was considered to be a approach to evaluate the caries risk^27^. The species-specific FISH and qPCR results showed D-Cys displayed a down-regulation effect on *S. mutans* and an up-regulation effect on *S. sanguinis* in dual-species biofilms. Besides, the ratio of *S. sanguinis* to *S. mutans* in multispecies biofilms increased significantly in D-Cys-treated groups compared to the control group. We speculated that the change in bacterial composition might be due to the different effects of D-Cys on the growth of these two strains. According to the result from the growth curve assay (Fig. 1), *S. mutans* displayed more sensitive than *S. sanguinis* to the D-Cys. D-Cys significantly inhibited *S. mutans* growth, while a slight growth inhibition occurred with D-Cys treatment in *S. sanguinis* group. D-Cys relieved the inhibition effect of *S. mutans* on *S. sanguinis*, thus making *S. sanguinis* more in dual-species biofilms. It has been pointed out that the early colonization of *S. sanguinis* and its elevated levels in the oral cavity could significantly delay the colonization of *S. mutans*^9^. Therefore, the elevation of the *S. sanguinis* proportion in dual-species biofilms by D-Cys indicated an ecological benefit of D-Cys in terms of caries prevention and control.

## Conclusion

In conclusion, our results indicated that D-Cys could inhibit the growth and cariogenicity of dual-species biofilms formed by *S. mutans* and *S. sanguinis* and altered the dual-species biofilms to a healthier condition. Therefore, D-Cys has potential use as a new drug for dental caries prevention and treatment. However, the biocompatibility of D-Cys needs to be tested in further study.

## Materials and Methods

### D-cysteine preparation

D-Cys (Shanghai Yuanye Biological Technology Co., Ltd.,China) were prepared at concentration of 20 mM, 40 mM, 60 mM^14^ in brain heart infusion (BHI) broth medium (Difco, Sparks, MD, USA).

### Bacteria inoculation, biofilm formation

*S. mutans* UA159 and *S. sanguinis* ATCC10556 were provided by the State Key Laboratory of Oral Diseases (Sichuan University, Chengdu, China). A single colony of *S. mutans* or *S. sanguinis* were precultured in BHI medium at 37 °C under anaerobic condition (80% N_2_, 10% CO_2_, 10% H_2_) overnight. The concentration of *S. mutans* and *S. sanguinis* was adjusted to 1 × 10^6^ CFU/mL in the study. BHI supplemented with 0.2% sucrose (BHIS) was used to support the biofilms formation^28–30^. For single-species biofilm formation, 200 µL of *S. mutans* or 200 µL of *S. sanguinis* were incubated in 96-well plates. For dual-species biofilm formation, 1 mL of *S. mutans* and 1 mL of *S. sanguinis* were incubated in 24-well plates. All the biofilms were cultured under anaerobic condition.

### Planktonic cell growth

For planktonic growth curve assays of *S. mutans* and *S. sanguinis*, bacterial cultures in exponential phase were added to BHI medium (without sucrose) containing different concentrations of D-Cys. Two hundred microliter of the bacteria with D-Cys were put into selected wells of a sterile 96-well microtiter plate and incubated at 37 °C (keep out of uncontaminated) for 24 h. The turbidity was measured by optical density (OD) at 600 nm using a microplate reader (SpectraMax 190; Molecular Devices, Inc., Sunnyvale, CA) every 1 h. There were six replicates of each bacterium for each D-Cys concentration.

### Crystal violet staining assay

Crystal violet staining assay was performed to measure the biofilm biomass. Two hundred microliter of the exponential-phase bacteria culture with D-Cys was cultured in the selected wells of a sterile 96-well microtiter plate for 24 h. Biofilms were rinsed by phosphate buffered saline (PBS) twice, then fixed by 100% methyl alcohol for 15 min. The biofilms on the bottom of the microplates were stained with 200 µL of 0.1% crystal violet for 5 min and then washed with sterile distilled water to remove the residual dye. The bounded crystal violet was released by 200 µL 95% ethanol. Before the measurement, the test elution liquid of crystal violet was diluted 1:4 with 95% ethanol to ensure that the readings were within the range of the spectrophotometer. The absorbance of released crystal violet in ethanol was recorded at OD_575 nm_ by a spectrophotometer. There were six replicates of each bacterium for each D-Cys concentration.

### MTT assay

MTT (3-(4,5-Dimethylthiazol-2-yl)-2,5-diphenyltetrazolium bromide) assay was performed to test the biofilms activity^31^. MTT solution was prepared in PBS at a working concentration of 0.5 mg/mL. Discs with 24 h dual-species biofilm were rinsed in PBS and transferred to a new 24-well plate supplemented with 1 mL of MTT solution, and then were incubated for 1 hour at 37 °C in 5% CO_2_ in the dark. An hour later, put each disc into a new 24-well plate, and added 1 mL of DMSO (Dimethyl sulfoxide) to cover the biofilm. Then the plate was incubated for 20 min at room temperature on an orbital platform in the dark (covered with foil). After incubation, 200 µL of DMSO solution was transferred to a 96-well plate and the absorbance was detected at OD_540 nm_ by a spectrophotometer. There were six replicates for each D-Cys concentration.

### Lactic acid production measurement

After incubation for 24 h, dual-species biofilms cultured on discs were washed with PBS to remove loose bacteria and then were transferred to a new 24-well plate with 1.5 mL buffered peptone water (BPW) accompanied with 0.2% sucrose^32^. Biofilms were incubated at 37 °C in 5% CO_2_ for 3 hours. Lactate concentrations were determined using an enzymatic (lactate dehydrogenase) method. 200 μL of collected BPW solutions were transferred to a new 96-well plate and were measured at the OD_340 nm_ with a microplate reader^33,34^. There were six replicates for each D-Cys concentration.

### Extracellular polysaccharide synthesis measurement by a confocal laser scanning microscope (CLSM)

Dual-species biofilms cultured on sterilized glass discs were grown in BHIS medium and supplemented with 2.5 μM Alexa Fluor 647-labeled dextran conjugate (Molecular Probes) for 24 h. Biofilms were washed with PBS for three times and stained with 2.5 μM SYTO 9 (Molecular Probes) for 15 min. The samples were imaged with a Leica DMIRE 2 confocal laser scanning microscope equipped with a 60× oil immersion objective lens. The 3-dimensional reconstruction was performed with Imaris 7.0.0 (Bitplane, Zürich, Switzerland). The quantification of EPS and bacteria volume was performed with Image-Pro Plus (Media Cybernetics, Silver Spring, MD, USA) and COMSTAT^35,36^. The EPS stained red color and each pixel site could be identified and quantified by this software. We calculated by counting the intensity and presence of color at each pixel site in each picture. In order to keep consistent between images, the parameters were set at beginning and each picture used the same parameters.

### Live/Dead assay

Dual-species biofilms cultured on discs were grown in BHIS medium for 24 h. Biofilms were washed with PBS for three times and stained with 2.5 μM SYTO 9 (Molecular Probes, Invitrogen) and propidium iodide (Molecular Probes) for 15 min. The samples were imaged with a DMIRE2 confocal laser scanning microscope (Leica, Wetzlar, Germany) equipped with a 60× oil immersion objective lens^37^. The quantification of live/dead was performed with Image-Pro Plus (Media Cybernetics, Silver Spring, MD, USA).

### Biofilms structure observation by a scanning electron microscope (SEM)

After culturing in BHIS medium for 24 h, the biofilms grown on sterilized glass discs were rinsed three times with PBS to remove loose planktonic bacteria and fixed with 2.5% glutaraldehyde overnight at 4 °C. Then, the discs were washed twice in sterile water (immersion in water per washed for 10 mins) and serial dehydrated with graded ethanol (50%, 70%, 75%, 80%, 85%, 90%, 95%, and 100%, immersion in ethanol per dehydrated for 10 mins). Then the samples were sputter-coated with gold for SEM imaging (Quanta 200, FEI, Hillsboro, OR, USA).

### Bacterial composition analysis in dual-species biofilms

The 24h dual-species biofilms grown on sterilized glass discs were washed with PBS for three times, fixed in 4% parafomaldehyde overnight, and investigated by species-specific fluorescent *in situ* hybridization (FISH) probes as previously described^38^. The labeled biofilms were imaged with an OLYMPUS FV1000 confocal laser scanning microscope (OLYMPUS, TOKYO, JAPAN) equipped with a 100× oil immersion objective lens. The bacterial composition was further quantified by species-specific real time quantitative polymerase chain reaction.

Total DNA of biofilms were extracted and purified using a TIANamp Bacteria DNA kit (TIANGEN, Beijing, China) followed by the manufacturer’s directions. The bacteria were lysed using enzymatic lysis buffer (20 mM Tris-HCl, pH 8.0; 2 mM sodium EDTA and 1.2% Triton X-100) containing 25 mg/mL of lysozyme at 37 °C for 1.5 h. The purity and concentration of DNA were detected by NanoDrop 2000 spectrophotometer (Thermo Scientific, Waltham, MA, USA). The quantitative polymerase chain reaction (qPCR) was used to quantify *S. mutans* and *S. sanguinis*. qPCR amplification was performed on the Bio-Rad CFX96 system (Bio-Rad, Hercules, CA, USA). The reaction mixture (25 μL) contained Premix Ex Taq (Takara Bio Inc, Shiga, Japan), template DNA, forward and reverse primers (10 mM each), and probes (10 mM). The sequences of probes were *S. mutans* (5′-FAM-TGGAAATGACGGTCGCCGTTATGAA-TAMRA-3′) and *S. sanguinis* (5′-FAM-TGTTCGGGCTCATGATA-Eclipse-3′). Quantification cycle (Cq) were determined, and the CFU/mL was calculated based on the standard curve (log CFU/mL versus Cq) generated using standard strain (*S. mutans* UA159 and *S. sanguinis* ATCC10556)^39,40^.

### Statistical analysis

Each experiment was independently repeated at least three times. One-way analysis of variance (ANOVA) was performed to detect the significance of the variables. Bartlett test was used before the ANOVA. Student Newman-Keuls test was used to compare the data at a *P* value of 0.05. Statistical analysis was performed with the SPSS software, version 17.0 (SPSS Inc., Chicago, IL, USA).

## Supplementary information

Dataset 1

## Data Availability

The datasets generated and analyzed during the current study are available from the corresponding authors on reasonable requests.
