# Supplementary material for: Effect of D-cysteine on dual-species biofilms of Streptococcus mutans and Streptococcus sanguinis
Source: Sci Rep. 2019 Apr 30;9:6689. doi: 10.1038/s41598-019-43081-1 (PMC6491432; doi:10.1038/s41598-019-43081-1)
Supplement: Supplementary file 1 — Dataset 1 [file 41598_2019_43081_MOESM1_ESM.docx]

Effect of D-cysteine on dual-species biofilms of *Streptococcus mutans* and *Streptococcus sanguinis*

Xiao Guo^1,2#^, Shiyu Liu^1,2^, Xuedong Zhou^1,2^, Hongying Hu^1,2^, Keke Zhang^3^, Xinmei Du^1^, Xian Peng^1^, Biao Ren^1^, Lei Cheng^1,2^*, Mingyun Li^1^*

**Appendix**

Quantitative polymerase chain reaction

The quantitative polymerase chain reaction (qPCR) was used to quantify the number of *Streptococcus mutans* and *Streptococcus sanguinis*. The qPCR amplification was performed on the Bio-Rad CFX96 system (Bio-Rad, Hercules, CA, USA). The reaction mixture (25 μL) contained Premix Ex Taq (Takara Bio Inc, Shiga, Japan), template DNA, forward and reverse primers (10 mM each), and probes (10 mM). The real-time PCR were performed as follows: 95°C for 3 min, followed by 40 cycles of 95°C for 10 s and 56°C for 30 s. The sequences of probes were *S. mutans* (5’-FAM-TGGAAATGACGGTCGCCGTTATGAA-TAMRA-3’) and *S. sanguinis* (5’-FAM-TGTTCGGGCTCATGATA-Eclipse-3’). Quantification cycle (Cq) were determined, and the CFU/mL was calculated based on the standard curve (log CFU/mL versus Cq) generated using standard strain. The *S. mutans* UA159 and *S. sanguinis* ATCC10556 were chosen to measure standard curves (Appendix Fig. 1). The standard curves were drafted for each probe set by using threshold cycle values obtained by amplifying successive 10-fold dilutions of known concentrations of DNA which stands for corresponding concentration of bacteria from 10^9^ CFUs to 10^4^ CFUs.


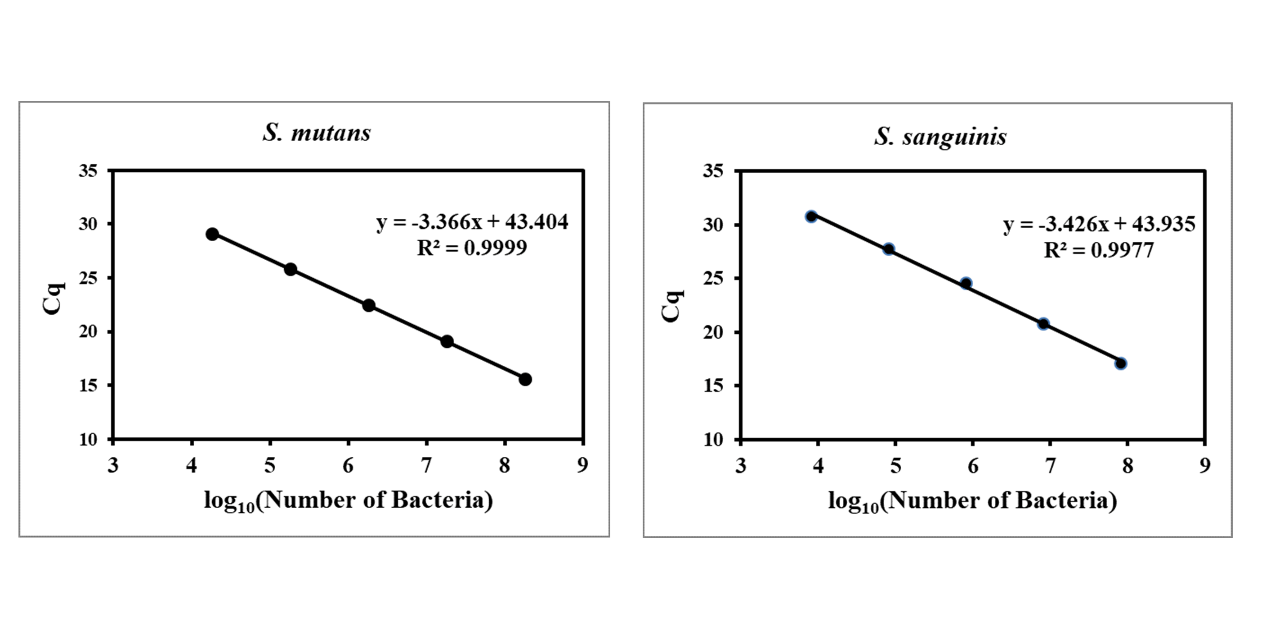


**Appendix Figure 1.** Standard curve generated by known numbers of *Streptococcus mutans* and *Streptococcus sanguinis*,
